# Design of a Magnetic Soft Inchworm Millirobot Based on Pre-Strained Elastomer with Micropillars

**DOI:** 10.3390/biomimetics8010022

**Published:** 2023-01-06

**Authors:** Yuzhang Wei, Zehao Wu, Ziyi Dai, Bingpu Zhou, Qingsong Xu

**Affiliations:** 1 Department of Electromechanical Engineering, Faculty of Science and Technology, University of Macau, Avenida da Universidade, Taipa, Macau, China; 2 Institute of Applied Physics and Materials Engineering, University of Macau, Avenida da Universidade, Taipa, Macau, China

**Keywords:** soft robot, magnetic actuation, bio-inspired robot, stretched elastomer, bionic inchworm, millirobot

## Abstract

Rather than using longitudinal “muscle” as in biological inchworm, the existing magnetic active elastomer (MAE)-based inchworm robots utilize magnetic torque to pull and push the soft body, which hinders its locomotion mobility. In this paper, a new pre-strained MAE inchworm millirobot with micropillars is proposed. The pre-strained elastomer serves as a pre-load muscle to contract the soft body, and the micropillars act as tiny feet to anchor the body during the locomotion. The proposed magnetic inchworm robot features a simple fabrication process that does not require special magnetization equipment. For the first time, the pre-load muscle is introduced in the design of magnetic inchworm robots, making it more like a real inchworm in terms of locomotion mechanism. The locomotion principle and parametric design for the desired locomotion performance have been investigated. Experimental results show that the fabricated magnetic inchworm robot (size: 10 mm × 5 mm, micropillars length: 200 µm, and mass: 262 g) can locomote on a smooth acrylic surface (roughness of 0.3 µm) at the speed of 0.125 body lengths per second, which is comparable with the existing magnetic inchworm robots. Moreover, the locomotion capabilities of the inchworm robot on wet surfaces and inclined planes have been verified via experimental studies.

## 1. Introduction

Conventional rigid-body robots have been widely used for delivering fast and precise motions. However, due to the bulky structure, difficult interaction in dynamic environments, and poor adaptability, it is hard or even impossible for rigid-body robots to accomplish some specific works, such as grasping different objects and walking through narrow curved paths [1]. Originally inspired by nature, soft robots (normally composed of elastomers and gels) exhibit the advantages of light-weight, high energy density, mechanical compliance, low cost, and biocompatibility [2]. These advantages endow soft robots with the merits of safe human–robot interaction while manipulating objects and adaptability to confined and dynamic environments. Soft robots exhibit great potential in soft exoskeletons, endoscopes, drug delivery, blood clot cleaning, and human assistance [3,4].

Biological inchworms (e.g., larva of Geometer moth) have been extensively studied as soft and rigid robots due to their simple and compliant structure. Inchworms move with an anchor push-pull strategy, i.e., anchoring their anterior leg and contracting the longitudinal muscles, pulling the posterior leg forward; anchoring the posterior leg and releasing the muscle, pushing the anterior leg forward and following the looping gait. Various inchworm-based soft robots have been developed with different actuators, such as dielectric elastomer (DE) [5], shape memory alloy (SMA) [6,7], liquid crystalline elastomer (LCE) [8], piezoelectric material (PEM) [9], twisted and coiled polymer (TCP) [10], and magneto-active elastomer (MAE) [11,12,13,14,15,16,17]. Among them, MAE-based inchworms exhibit the advantages of strong load-carrying ability, small scale, flexibility, adaptability to harsh environments, and untethered structures [11,12,13,14,15,16,17], making them the closest to the biological inchworm. Moreover, the magnetic field is easy to be decoupled from other stimuli, and the spatial gradients are intrinsic [18,19]. Thus, the MAE materials have been applied in crawling robots [20], swimmers [21], grippers [22], micropumps [23], and bionic robots [11].

MAE materials for soft robots are desired with robust toughness, tear resistance, and low elastic modulus. The most popular fabrication method is to mix magnetic micro/nano-particles (soft or hard magnetic particles) with elastomer [1,24]. It is notable that 3D printing technology emerges as an effective method for developing complex MAE structures. Actually, hard magnetic particles (e.g., NdFeB) can maintain a given magnetization when no external magnetic field is applied, and vice versa for soft magnetic particles (e.g., Fe_3_O_4_). Generally, hard magneto-active elastomer (hMAE) can change its shape fast, while soft magneto-active elastomer (sMAE) can achieve a large reversible volume [25]. With continuously distributed magnetization (or programmed magnetization) and a controlled magnetic field, the hMAE can obtain complex movements, such as jumping, crawling, gripping, and releasing, making it an ideal option for developing bionic robots [17]. MAE-based inchworms can mainly be divided into three groups according to the modalities of magnetic materials, i.e., magnetic blocks [15], magnetic filament [11,16], and magnetic particles [12,14]. In the literature, Joyee and Pan developed a 3D-printed inchworm robot with two permanent magnetic blocks at each end, serving as the two legs of the inchworm [15]. However, the rigid part of the magnetic blocks will cause relatively large contact pressure and may induce damage to the crawling surface. The embedded aligned magnetic filament stiffens the soft structure, and the magnetization is also fixed, limiting its application in the biological area. Actually, the magnetic particles are uniformly distributed into elastomer, and the magnetization can be well adjusted by uni-axial magnetization [26], continuously distributed magnetization [14,27], and programmed magnetization [28]. The magnetic particle-based MAE inchworm robot outperforms the others in terms of softer structure, smaller size, and better flexibility.

Currently, the MAE-actuated inchworm robots (based on magnetic particles) are mostly actuated by electromagnetic coils [14] or permanent magnets [12]. The former has a confined workspace with complex mechanical and control systems. The latter permanent magnet-actuated MAE is more preferable for bionic magnetic inchworm robots. The existing magnetic inchworm robots require controlled magnetization or special 3D printing technology with custom-made or expensive instruments [11,12,14,16,27], which impose difficulties in fabricating the simple-structure robot. Moreover, from the bionic perspective, the current magnetic inchworm robots are only constructed by magnetic sheets without legs and are driven by magnetic torques rather than the longitudinal muscle. By contrast, a biological inchworm utilizes longitudinal muscle to contract and release its body, with one leg fixed and the other leg moving with the body. Thus, the magnetic inchworm robot is expected to have easy fabrication and be more like a biological inchworm in terms of similar structures with legs and longitudinal muscle.

To this end, an easily-fabricated bionic magnetic inchworm robot composed of pre-strained MAE with micropillars is presented in this work. The pre-strained MAE is bonded with Polydimethylsiloxane (PDMS) substrate using double-sided tape, acting as a pre-load “muscle” to recover the arched shape when no magnetic field is applied. The pre-load muscle contracts the magnetic inchworm robot body to make the posterior section move forward when the anterior leg is fixed with micropillars by magnetic force. The magnetic micropillars are adopted as tiny legs to anchor the surface, like the biological inchworm gripping the ground surface. In this way, magnetization is not necessary for actuation, which simplifies the manufacturing process.

The main contribution of this work is the design of a new bionic magnetic inchworm robot. The robot adopts micropillars as bionic legs to anchor on the ground and uses pre-strained elastomer as the longitude muscle to contract the robot body, rather than a simply magnetized sheet, as used in the literature. The remaining parts of the paper are organized as follows. In Section 2, the new magnetic inchworm robot is designed and fabricated. The robot parameters governing the locomotion performance are analyzed in Section 3. Section 4 presents the experimental results of the locomotion test for the fabricated magnetic inchworm robots. The conclusion is summarized in Section 5.

## 2. Design and Fabrication of a MAE-Based Inchworm Millirobot

### 2.1. Concept Design

To create a bionic inchworm vividly, the magnetic inchworm robot should have a simple, soft, and untethered body with two legs and longitudinal muscles. Concerning the soft and untethered body, MAE outperforms SMA, DE, PEM, and TCP, which are either tethered or contain bulky hard components. Although LCE is a promising soft untethered material, the low efficiency of phase transition limits its movement efficiency. Thus, MAE is selected as the main body of the magnetic inchworm robot owing to its ability for miniaturization, in addition to its characteristics of soft and untethered properties.

Unlike the active longitudinal muscles in SMA, DE, PEM, and LCE, the elastic restoring force of a stretched MAE is adopted as the strength of pre-load longitudinal muscle. Regarding the two legs for grabbing the ground, inspired by Lu and co-workers [26], micropillars are chosen to anchor the soft body. It is notable that isotropic MAE without magnetization is introduced to simplify the fabrication process and eliminate the requirement for extra equipment.

### 2.2. Fabrication Process

**Figure 1:**
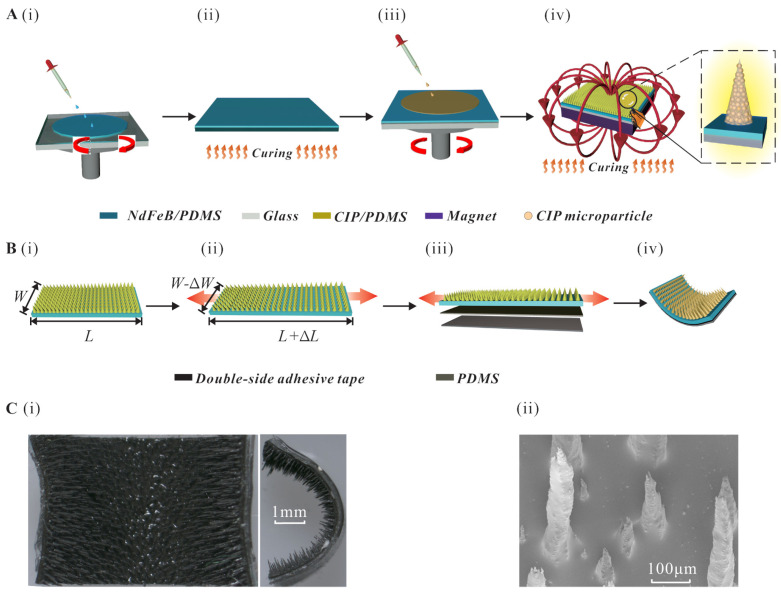
Fabrication process of the bionic magnetic inchworm robot. (**A**) Fabrication process of MAE with micropillars. (**i**) The NdFeB/PDMS composite is spin-coated onto a glass substrate to form the first layer of the MAE. (**ii**) The composite is cured under 150 °C for 20 min. (**iii**) CIP/PDMS composite is spin-coated onto the first layer. (**iv**) The substrate is moved onto a permanent magnet (∼400 mT) and cured under 40 °C for 4 h. (**B**) Fabrication process of proposed bionic magnetic inchworm robot. (**i**) A piece of elastomer is cut from the fabricated MAE. (**ii**) The elastomer is stretched to length Δ*L* with two ends fixed with two precise linear stages. (**iii**) Double-sided adhesive tape and PDMS substrate are spread onto the stretched MAE in sequence. (**iv**) Schematic diagram of the fabricated magnetic inchworm robot. (**C**) Optical image of the proposed bionic magnetic inchworm robot. (**i**) Microscope view of the fabricated magnetic inchworm robot. (**ii**) SEM image of the micropillars.

Based on the concept design, the micropillars-based stretched MAE inchworm robot has been fabricated. The fabrication process of MAE with micropillars is shown in Figure 1A. To start with, a NdFeB/PDMS composite (weight ratio of PDMS, curing agent and NdFeB (∼5 µm in average size) as 15:1:10) is spin-coated (900 rpm for 20 s) onto a clean glass substrate with a side length of 5 cm (see Figure 1A(i)). Then, a curing process under 150 °C is then carried out for 20 min to ensure the complete solidification of the membrane (see Figure 1A(ii)). Following that, another membrane of the carbonyl iron powder (CIP) (0.2–2 µm in diameter)/PDMS composite is spin-coated (3250 rpm for 20 s, with a weight ratio of PDMS, curing agent and CIP as 15:1:10) onto the cured NdFeB/PDMS membrane (see Figure 1A(iii)). After that, the substrate is then moved to the surface of the squared permanent magnet (∼400 mT). Due to the existence of an external magnetic field, the uncured CIP/PDMS composite would instantly form the micropillar morphologies, as depicted in Figure 1A(iv). Note that the applied magnetic field is not strong enough to generate magnetization [29], and the length of micropillars is positively associated with the volume of CIP microparticles. Afterward, the glass substrate, along with the permanent magnet, is then totally moved to the oven (40 °C) for curing with a duration of 4 h. A piece of rectangle MAE is cut from the fabricated squared MAE with a specific length and width (see Figure 1B(i)). Then, the two ends of the piece of MAE are fixed by two precise stages (model: CA92714, Newport CO.) and stretched with a specific length (see Figure 1B(ii)). Following that, a double-sided adhesive tape (thickness: 150 µm, model: G4000, from Dexerials Co., Tokyo, Japan ) and PDMS (thickness: 150 µm) are spread on the stretched elastomer (see Figure 1B(iii)). After cutting the redundant parts, the magnetic inchworm robot is obtained (see Figure 1B(iv)). The microscope view of the magnetic inchworm robot and the scanning electron microscope (SEM) view of the micropillars are shown in Figure 1C(i,ii).

### 2.3. Locomotion Principle

**Figure 2:**
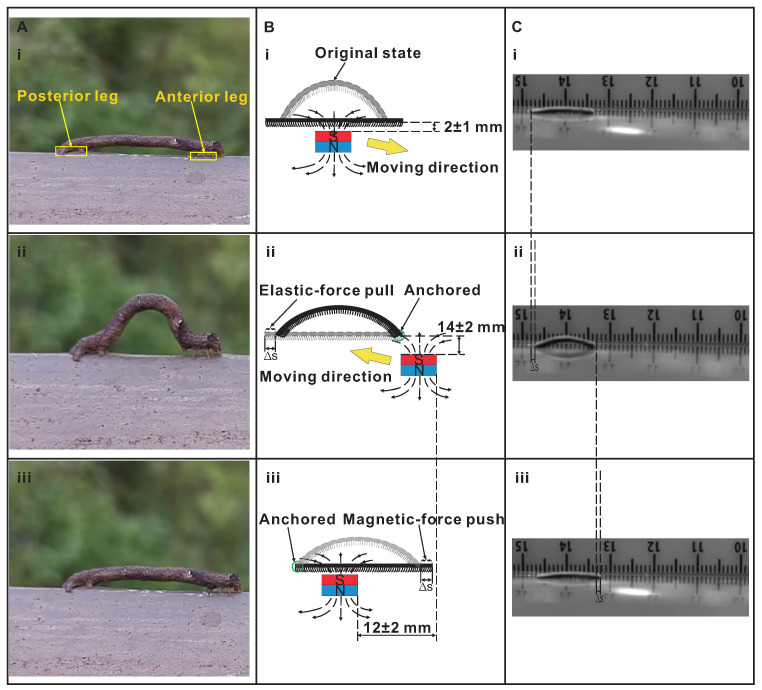
Illustration of inchworm robot locomotion. (**A**) Biological inchworm locomotion in a looping gait. (**i**) Initial position of the inchworm. (**ii**) The inchworm anchors its anterior leg and pulls its posterior leg moving forward. (**iii**) The inchworm anchors its posterior leg and pushes its anterior leg going forward. (**B**) Schematic diagram of motion decomposition of magnetic inchworm robot, and the gray shape and the black shape are undeformed and deformed shapes, respectively. (**i**) Permanent magnet is located below the curved magnetic inchworm robot (∼2 mm in vertical distance), and the attractive magnetic force makes it flatten. (**ii**) The magnet moves to the lower right (∼14 mm in vertical distance), and the magnetic attraction will anchor the anterior leg, and the pre-load elastic muscle will contract to pull the posterior leg moving forward. (**iii**) The magnet moves to the upper left (∼12 mm in horizontal distance), closer to the posterior leg, and the magnetic attraction will anchor the posterior leg and meanwhile attract the magnetic body to become flat, pushing the anterior leg forward. (**C**) Locomotion of fabricated magnetic inchworm robot. Locomotion in (**i**–**iii**) correspond to those in (**B**(**i**)–(**iii**)).

As shown in Figure 2A, the inchworm moves forward by anchoring the anterior leg and pulling the posterior leg forward while contracting the middle longitudinal muscles in sequence; anchoring the posterior leg and pushing the anterior leg while releasing the muscles in order. The designed magnetic inchworm robot aims at intimating the locomotion of an inchworm by magnetic force and elastic force. The driving dynamic magnetic field belongs to a spatial gradient, which is inherently generated by a moving cylindrical permanent magnet (∼500 mT at polar surfaces, diameter: 30 mm, and length: 30 mm).

The qualitative explanation of the locomotion principle is given as follows. First, the original state (i.e., curved state) is flattened by the magnetic attraction when the permanent magnet is put under the middle of the magnetic inchworm robot (∼2 mm in vertical distance) (see Figure 2B(i)). Second, when the permanent magnet moves to the lower right (∼14 mm in vertical distance) (moving direction shown in Figure 2B(i)), the anterior leg of magnetic inchworm robot anchors due to stronger magnetic attraction. Meanwhile, the weaker magnetic attraction on the posterior leg makes the elastic restoring force of the stretched MAE prevail. It serves as the pre-load longitudinal muscle to contract and then pull the posterior leg to move forward (see Figure 2B(ii)). Note that the way of movement for permanent magnet aims at anchoring the anterior leg and, meanwhile, reducing the magnetic influence on the posterior leg. Third, the permanent magnet moves to the upper left (∼12 mm in horizontal distance), closer to the posterior leg. Meanwhile, the magnetic attraction will anchor the posterior leg and attract the magnetic body to flatten, pushing the anterior leg to move forward (see Figure 2B(iii)). The corresponding movements of the fabricated magnetic inchworm robot are shown in Figure 2C(i–iii).

## 3. Parametric Analysis of the Millirobot Design

To design the magnetic inchworm robot, it is necessary to investigate the influence of the dominant parameters of the robot on the locomotion performance. In particular, the length-to-width ratio, length of micropillars, and stretched length parameters are studied by fabricating a series of millirobots with different sets of parameters. For illustration, the original length of the MAE film is selected as 14 mm, including an extra section (4 mm) for clamping in stretched equipment, and the lengths of double-sided adhesive tape and PDMS film are set as 10 mm.

First, the length-to-width ratio is determined by experimental testing. Specifically, the robots with three length-to-width ratios of 10/2.5, 10/5, and 10/7.5 are fabricated and tested. The purpose is to determine a feasible inchworm robot to analyze the effects of stretched length and the length of micropillars. The experimental results show that the magnetic inchworm robot with the length-to-width ratio of 10/5 can move like an inchworm crawling mechanism, whereas the robots with the other two length-to-width ratios can only be dragged forward by magnetic force because the friction is too small to anchor and the elastic force is too big to deform under length-to-width ratios of 10/2.5 and 10/7.5, respectively. Note that the ratio of 20/10 is used in [12], which serves as a reference for the length-to-width ratio selection. Hence, the robot with a length-width ratio of 10/5 is chosen to provide a feasible platform for analyzing the two other important parameters, i.e., stretched length and the length of micropillars.

**Figure 3:**
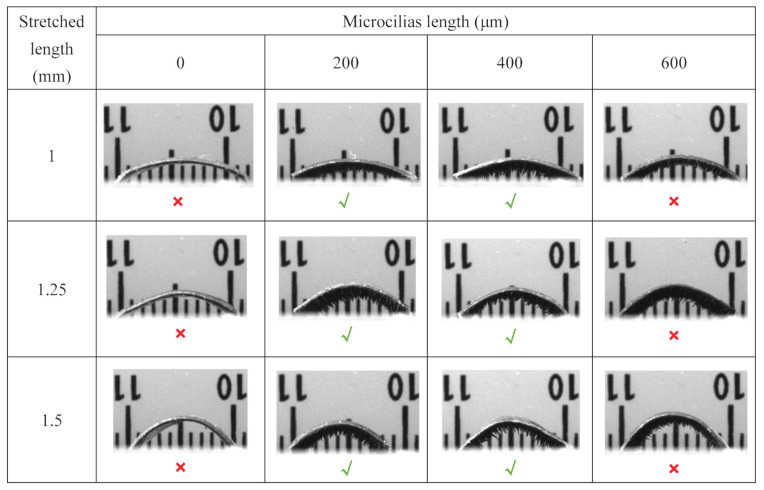
Performance evaluations of magnetic inchworm robots for combinations with stretched length set and micropillar length set. ✖ means that the magnetic inchworm robot cannot move like a biological inchworm, and ✔ indicates that the inchworm robot can move like a biological inchworm crawling mechanism.

In particular, the effects of stretched length and length of micropillars are tested as follows. To verify the effectiveness of micropillars in locomotion and to determine the suitable length of micropillars, millirobots with different micropillar lengths (0, 200 µm, 400 µm, and 600 µm) are considered. Here, the length of micropillars describes the mean value of multiple micropillars for a specific robot and the length is positively associated with the volume of CIP microparticles. In addition, the original length of the magnetic inchworm robot is stretched by 1.00, 1.25, and 1.50 mm, which correspond to three arched inchworm robots with obviously different curvatures. A series of inchworm robots with different combinations of the design parameters, as mentioned above have been fabricated, as shown in Figure 3. The locomotion performances have been evaluated by conducting experimental tests to verify whether the fabricated magnetic inchworm robots can move with a biological inchworm crawling mechanism.

To verify the necessity of the micropillars, three magnetic inchworm robots without micropillars are fabricated, as shown in column 1 of Figure 3. It is found that the robots without micropillars cannot move like inchworm crawling mechanisms, whereas the robots with micropillars (e.g., lengths of 200 and 400 µm in columns 2 and 3 of Figure 3) can move like a real inchworm. The reason lies in that the strong adhesion of PDMS in the substrate prevents the micropillars-free robot from moving forward. Meanwhile, the micropillars enable the separation between the substrate and robot body and provide certain friction. Moreover, the elastic deformation of the micropillars increases the coefficient of friction, assisting in anchoring the anterior leg during the locomotion. As a result, the magnetic inchworm robots with micropillars can move like an inchworm under permanent magnet actuation and elastic force action.

Concerning the length of micropillars, the magnetic inchworm robots with micropillar lengths of 200 and 400 µm can move like biological inchworm’s crawling mechanism, whereas the robots with a micropillar length of 600 µm cannot move, as illustrated in Figure 3. This phenomenon indicates that the movement of the anterior leg must overcome the elastic force generated by the deformed micropillars and the friction to enable the locomotion of the robot. When the length of the micropillars is too long, the magnetic attraction force is smaller than the elastic force and friction force, resulting in no movement. Note that the movement of the magnetic inchworm robot is also governed by the coefficient of friction of the contact surface. In other words, the magnetic inchworm robot has different locomotion performances (e.g., step size and speed) under different surface roughness conditions.

Regarding the stretched length, the experimental results show that all of three sets of magnetic inchworm robots can move like real inchworms. However, different stretched lengths produce different curvatures for the robots. As a consequence, the elastic restoring forces are different, inducing different pre-load strains (see Figure 2B(ii)). According to the test results of magnetic inchworm robots with three stretched lengths, the robot with a 1.00 mm stretched length produces a smaller step size, while the robot with a stretched length of 1.25 mm exhibits a larger step size. The robot with a stretched length of 1.50 mm offers a smaller step size than that of 1.25 mm. The reason lies in that the relatively large curvature generates too large of an elastic restoring force to be overcome (see Figure 2B(i)) and exhibits a large contact angle and high center of gravity, which cause difficulty and instability in anchoring the anterior leg.

## 4. Experimental Results and Discussion of Millirobot Locomotion

To evaluate the locomotion performance of magnetic inchworm robots as illustrated in Figure 3, these millirobots are tested for locomotion on an acrylic plane surface with a roughness of 0.3 µm. The inchworm robot is sensitive to the position and speed of the actuated permanent magnet, and hence, the permanent magnet is manually carried to move up and down diagonally, limited by the usable equipment. The movement speed of the magnet is approximately 18 ± 2.8 mm/s with the horizontal movement distance of 12 ± 2 mm and the vertical movement distance of 14 ± 2 mm. Considering the inevitable deviation for manual operation, the main parameter concerned is the maximum step size that the robot can achieve beyond the deviation of manual operation. Note that the movement speed should not be too high. Otherwise, the robot will turn over or be directly dragged away.

**Figure 4:**
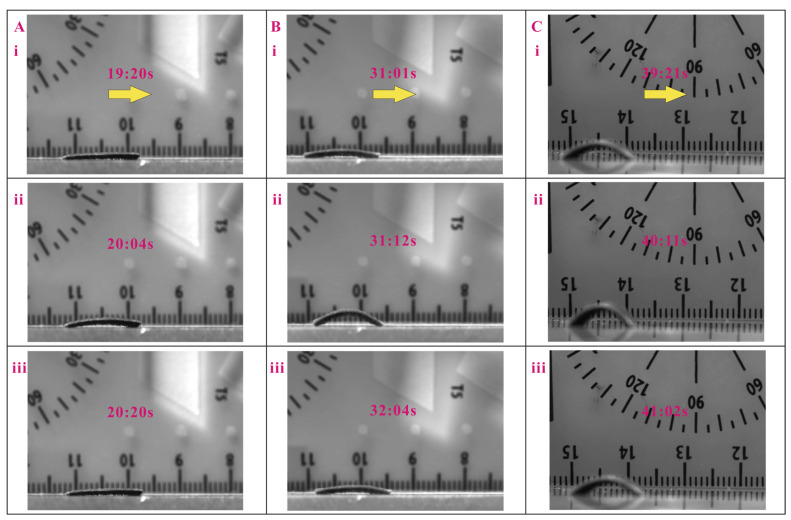
Locomotion test results of magnetic inchworm robots with 200 µm micropillars. (**A**) Stretched length is 1 mm, and the step size is about 0.33 mm. (**B**) Stretched length is 1.25 mm, and the step size is about 1.25 mm. (**C**) Stretched length is 1.5 mm, and the step size is about 0.83 mm. In each subfigure, the yellow arrow represents the movement direction. i, ii, and iii indicate the snapshots at different times.

**Figure 5:**
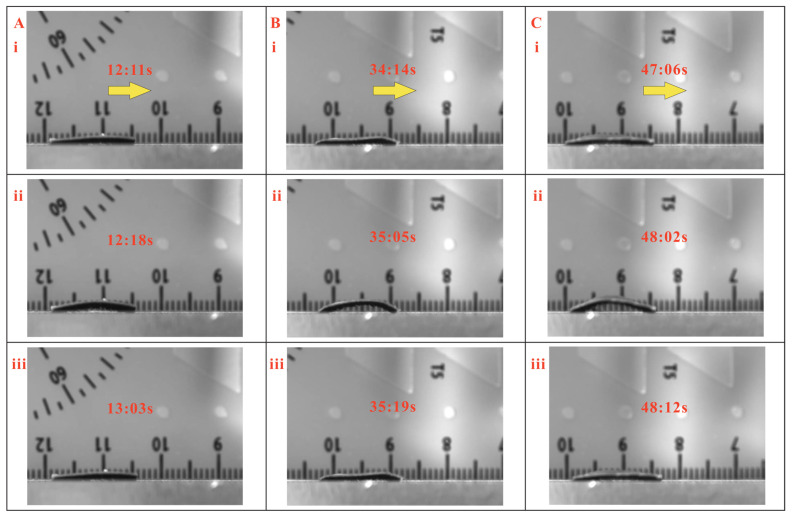
Locomotion test results of magnetic inchworm robots with 400 µm micropillars. (**A**) Stretched length is 1 mm, and the step size is about 0.39 mm. (**B**) Stretched length is 1.25 mm, and the step size is about 1 mm. (**C**) Stretched length is 1.5 mm, and the step size is about 1 mm. In each subfigure, the yellow arrow represents the movement direction. i, ii, and iii indicate the snapshots at different times.

Multiple experiments have been conducted, and the full demonstrations of locomotion cycles for 5 mm displacement of the magnetic inchworm robots with micropillar lengths of 200 µand 400 µm are given in Supplementary Videos S1 and S2. The video snapshots of one locomotion cycle of three magnetic inchworm robots with the same micropillars of 200 µm and different stretched lengths of 1.00, 1.25, and 1.50 mm are shown in Figure 4. In addition, the test results of the three magnetic inchworm robots with the same micropillars of 400 µm are depicted in Figure 5. The experimental results indicate that the proposed magnetic inchworm robots can successfully imitate the movement mechanism of biological inchworms with the anchoring pull-and-push strategy.

In view of the comparison of different stretched lengths, the magnetic inchworm robots with a stretched length of 1.25 mm produce a larger step size (see Figure 4B) than the millirobots with stretched lengths of 1.00 mm and 1.50 mm. Hence, the stretched length of 1.25 mm enables a higher efficiency of locomotion. Concerning the length of micropillars, it mainly affects the coefficient of friction and the elastic restoring force of the deformed micropillars. The magnetic inchworm robots with shorter micropillars are suitable for moving on rough surfaces, whereas the millirobots with longer micropillars are more appropriate for moving on smooth surfaces.

**Figure 6:**
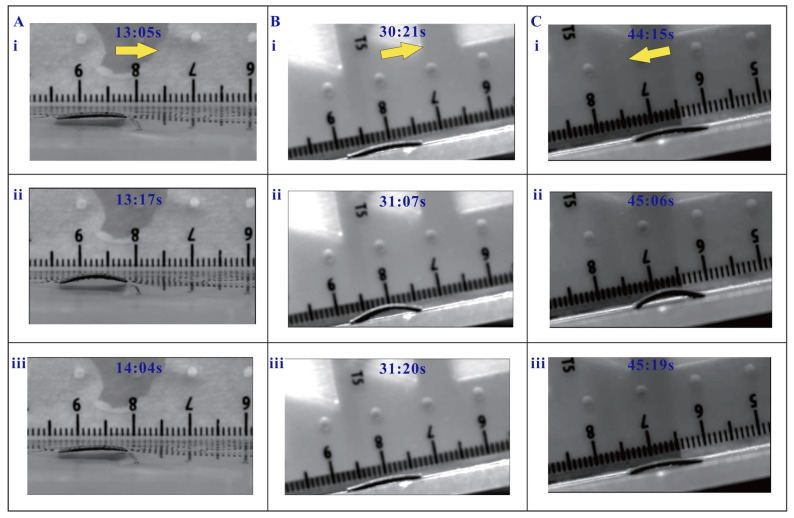
Locomotion test results of magnetic inchworm robot in special conditions. (**A**) Locomotion test in water with a level height of 1 mm. (**B**) Upward locomotion in an inclined plane with 10° inclination angle. (**C**) Downward locomotion in an inclined plane with 10° inclination angle. In each subfigure, the yellow arrow represents the movement direction. i, ii, and iii indicate the snapshots at different times.

Moreover, the proposed magnetic inchworm robot with a 1.25 mm stretched length and 200 µm micropillars has been tested to successfully move on wet surfaces with a water level height of 1 mm, and on an inclined plane surface with an inclination angle of 10°. The experimental results are shown in Figure 6 and Supplementary Video S3. It is notable that the friction forces on the surfaces in wet conditions and inclined planes for upward locomotion are smaller than those in flat plane surface locomotion, and the opposite is true for downward locomotion. Therefore, the step sizes of the millirobots for locomotion on wet surfaces and upward locomotion on inclined planes are smaller than that of the millirobot with downward locomotion on an inclined plane (see Figure 6).

Considering that the mass, surface roughness, and body length considerably influence the speed of the magnetic inchworm robot, a comparison study versus existing magnetic inchworm robots has been conducted, as tabulated in Table 1. As shown in Supplementary Video S1, the inchworm robot (1.25 mm stretched length and 200 µm micropillars) moves a distance of 6 mm within 4 s. As compared with existing designs, the proposed magnetic inchworm robot can move on a smoother surface with similar self-mass and movement speed (considering the speed of the control mechanism). The main advantages of the proposed magnetic inchworm robot lie in the simplification of the fabrication and bionic similarity to real inchworms. The reported magnetic inchworm robot does not need extra special complex equipment to magnetize for fabrication. Actually, a square-shaped permanent magnet (about 400 mT) can be used to produce the micropillars. Like a biological inchworm using two legs to anchor and longitudinal muscle to complete pull-push motion, the proposed magnetic inchworm robot utilizes multi-micropillars to fix the two ends, and employs the elastic restoring force of pre-load longitudinal muscle and the magnetic force to achieve similar locomotion.

In practice, assembly errors are inevitably avoided during the fabrication, causing slight differences in the performance for the same series of fabricated millirobots. The main reason for the different performance is the manually inconsistent cutting of the double-sided adhesive tape and MAE. If they are cut in a standard pattern, the performance can be more consistent. Here, the function of anchoring is mainly achieved by magnetic attraction and elastic deformation of the micropillars. Nevertheless, this method requires careful movement control to anchor the anterior leg and, meanwhile, to prevent it from turning over or directly dragging away. In future work, the performance of the proposed magnetic inchworm robot will be further improved by specially designing both ends of the robot body to enhance the anchoring performance toward better locomotion on different surfaces.

## 5. Conclusions

In this paper, a new magnetic inchworm robot based on stretched elastomers and micropillars is presented. The stretched elastomer acts as pre-loaded muscle to contract to make the posterior leg move forward, similar to the longitudinal muscle in a real biological inchworm. The micropillars can separate the stick PDMS substrate from the contact surface and increase the coefficient of friction, contributing to the anchoring of one leg when moving forward. Analysis of the locomotion principle and influence of key design parameters of the magnetic inchworm robot on its locomotion performance has been performed. The experimental results show the fabricated magnetic inchworm robot can successfully imitate the locomotion strategy of a biological inchworm. As compared with existing magnetic inchworm robots, the proposed design (size of 10 mm × 5 mm, micropillars of 200 µm, and mass of 262 g) can move on smoother surfaces with similar mass and speed. The presented design enables a simpler fabrication process and is more like an inchworm in locomotion mechanism by using pre-load muscle and anchoring micropillars. Moreover, the proposed low-cost magnetic inchworm robot imposes no need for special magnetization for fabrication.

In future work, there are mainly three issues to be addressed. First, modeling the locomotion mechanism will be more convincing in proving the effectiveness of the micropillar in generating the asymmetric coefficient. Next, experiments will be carried out to measure the coefficients of friction with different sets of micropillars. Finally, intelligent control and standard fabrication process will be conducted to evaluate the reproducibility of the inchworm robot.

## Figures and Tables

**Table 1:** Performance comparison of existing magnetic inchworm robots and the proposed one.

Mass (g)	Surface Roughness (µm)	Relative Speed (Body Length/s)	Control Mechanism And Its Speed
0.167 [12]	Unknown	1.1	Magnet (431 mm/s)
0.77 [13]	1.2	0.14	Electromagnetic coils
Unknown [14]	Jagged grooves	0.025	Electromagnetic coils
0.2 [15]	2.5	0.125	Magnet (Unknown speed)
0.262 (This work)	0.3	0.125	Magnet (18 mm/s) and elastic force

## Data Availability

Not applicable.

## Supplementary Materials

The following supporting information can be downloaded at: https://www.mdpi.com/article/10.3390/biomimetics8010022/s1, Video S1: Locomotion demonstration of inchworm robots with the micropillar length of 200 micrometers; Video S2: Locomotion demonstration of inchworm robots with the micropillar length of 400 micrometers; Video S3: Locomotion demonstration of an inchworm robot in water.
